# Supplementary material for: Content-rich biological network constructed by mining PubMed abstracts
Source: BMC Bioinformatics. 2004 Oct 8;5:147. doi: 10.1186/1471-2105-5-147 (PMC528731; doi:10.1186/1471-2105-5-147)
Supplement: Additional File 2 — The original results of the above study (non-essential files are deleted to keep the file size under the limit set by BMC bioinformatics). [file 1471-2105-5-147-S2.bz2 › chilibotAdditionalFile2/dip50/378ID9927421E9791/html/NGFR_NTRK2.html]

 


 **NGFR** and **NTRK2** 
  
Found 102 abstracts in PubMed, retrieved 50.  
 

 What does Google say? 
 PDF only 
| .edu only 

---

**Interactive relationship** (e.g. stimulation, inhibition, etc)

**Inhibitory relationship**- Inhibition of p75NTR  [ **NGFR** ]  does not detectably reduce LTP or affect presynaptic function, but analyses of newly generated trkB  [ **NTRK2** ]  mutants implicate TrkB  [ **NTRK2** ] .  Ref: 10995833 J Neurosci, 2000
- The lack of covariation between p75NTR  [ **NGFR** ]  and trkB  [ **NTRK2** ]  and trkC regulation in aged rats indicates a changed role for p75NTR  [ **NGFR** ]  in senescent motoneurons.  Ref: 10350634 Brain Res Mol Brain Res, 1999
**Neutral relationship**- By immunohistochemistry, p75NTR  [ **NGFR** ]  alone was strongly expressed in TUNEL Bcl2 keratinocytes of the regressing outer root sheath, but both p75NTR  [ **NGFR** ]  and TrkB  [ **NTRK2** ]  and or TrkC were expressed by the nonregressing TUNEL Bcl2 secondary hair germ keratinocytes.  Ref: 11023977 FASEB J, 2000
- Although neurotrophins did not regulate p75NTR  [ **NGFR** ]  processing, the alpha and gamma secretase mediated cleavage of p75 is modulated by receptor tyrosine kinases Trks TrkA and TrkB  [ **NTRK2** ]  but not TrkC.  Ref: 12843241 J Neurosci, 2003

**Non-interactive relationship** (e.g. studied together, co-existance, homology, etc.)

- This is coupled to the ectopic expression of trk B  [ **NTRK2** ]  and trk C, as well as to the continued expression of trk A, and the loss of expression of p75NTR  [ **NGFR** ]  receptors.  Ref: 11027413 Prostate, 2000
- We have here examined the expression of neurotrophin receptor p75NTR  [ **NGFR** ] , trkA, trkB  [ **NTRK2** ] , and trkC mRNA and protein in intact and axotomized primary sensory neurons of young adult 3 months and aged 30 months rats.  Ref: 10404406 J Comp Neurol, 1999
- We show immunoreactivity to antibodies to nerve growth factor NGF, brain derived neurotrophin BDNF, neurotrophin 3 NT 3, neurotrophin 4 5 NT 4 5, the low affinity nerve growth factor receptor  [ **NGFR** ]  p75 and the high affinity tyrosine kinase receptors Trk B  [ **NTRK2** ]  and C in hepatic stellate cells and weak reactivity for BDNF, NT 3, and NT 4 5 in hepatocytes, in cryosections of human and rat liver, in normal and varying pathologic conditions.  Ref: 11124831 Hepatology, 2001
- Retrograde transport of neurotrophins from the eye to the brain in chick embryos roles of the p75NTR  [ **NGFR** ]  and trkB  [ **NTRK2** ]  receptors.  Ref: 8622129 J Neurosci, 1996
- After axotomy, a down regulation of mRNA and protein levels was observed for all neurotrophin receptors p75NTR  [ **NGFR** ] , trkA, trkB  [ **NTRK2** ]  and trkC in both young adult and aged rats.  Ref: 10404406 J Comp Neurol, 1999
- We have studied the expression of different splice forms of trkB  [ **NTRK2** ]  and trkC as well as p75  **NGFR**  in rat and mouse cochlea within this critical developmental period, using in situ hybridization, PCR, Northern blotting, and immunohistochemical analyses.  Ref: 10494076 J Comp Neurol, 1999
- The prenatal development of the neurons immunoreactive for high affinity tropomycin related kinase trk receptor pan trk which recognizes trkA, trkB  [ **NTRK2** ] , and trkC and low affinity p75 neurotrophin receptor p75NTR  [ **NGFR** ]  was examined in the human brain from embryonic weeks 10 to 34 of gestation.  Ref: 8761930 J Comp Neurol, 1996
- We show that NT2 N cells express rRNAs for TrkA, TrkB  [ **NTRK2** ] , and TrkC tyrosine kinase receptors and the low affinity nerve growth factor receptor  [ **NGFR** ]  p75NTR  [ **NGFR** ] .  Ref: 10386964 J Neurochem, 1999
- Finally, an expression of trkB  [ **NTRK2** ] .T1 and trkB  [ **NTRK2** ] .T2 in oligodendrocytes, full length trkB  [ **NTRK2** ]  and trkC in nerve fibers, and p75  **NGFR**  in Schwann cells was noted at the glial interface of the VIIIth nerve during the establishment of the glial transition zone.  Ref: 10494076 J Comp Neurol, 1999
- The specimens were analyzed immunohistochemically for neurotrophins nerve growth factor, brain derived neurotrophic factor, neurotrophin 3, neurotrophin 4 5 and their receptors TrkA, TrkB  [ **NTRK2** ] , TrkC, p75NTR  [ **NGFR** ] .  Ref: 11442674 Int J Urol, 2001
- Virtually all adult CSN express mRNA coding for the NT 3 receptor TrkC and the BDNF receptor TrkB  [ **NTRK2** ] , and after axotomy, CSN also express mRNA for the common neurotrophin receptor p75NTR  [ **NGFR** ] , suggesting a role of endogenous neurotrophins for survival regulation of CSN.  Ref: 11236067 Rev Neurosci, 2001
- Neurotrophin signaling is mediated by Trk family tyrosine kinases such as TrkA, TrkB  [ **NTRK2** ]  and TrkC, as well as by the pan neurotrophin receptor p75NTR  [ **NGFR** ] .  Ref: 11836532 Nat Neurosci, 2002
- Sections of pancreatic cancer and normal pancreatic tissue were examined by immunohistochemistry for the expression of the neurotrophins NGF, BDNF, NT 3, NT 4, and their receptors TrkA, TrkB  [ **NTRK2** ] , and TrkC, as well as the low affinity receptor, p75NTR  [ **NGFR** ] .  Ref: 11561004 J Histochem Cytochem, 2001
- We have studied the expression of p75NTR  [ **NGFR** ] , trkB  [ **NTRK2** ]  and trkC, respectively, in lumbar motoneurons of young adult 2 3 months and aged 30 months rats subjected to sciatic transection using in situ hybridization and immunohistochemistry.  Ref: 10350634 Brain Res Mol Brain Res, 1999
- With BDNF TrkB  [ **NTRK2** ]  promoting survival and NT 3 TrkC p75NTR  [ **NGFR** ]  promoting death, CSN integrate at least three different neurotrophin receptor signals for death survival decisions.  Ref: 11236067 Rev Neurosci, 2001
- METHODS Immunocytochemistry and Western blot analyses were performed using primary antibodies raised against NTs nerve growth factor NGF, brain derived neurotrophic factor BDNF, NT 3, and NT 4 5 and NT receptors TrkA, TrkB  [ **NTRK2** ] , TrkC, and p75NTR  [ **NGFR** ] .  Ref: 11053307 Invest Ophthalmol Vis Sci, 2000
- The neurotropins are survival and differentiation factors in the nervous system, acting via high affinity receptors of the trk protooncogene family and the low affinity p75 nerve growth factor receptor  [ **NGFR** ] , and have also been described in the rodent ovary, where changes in NT4 TrkB  [ **NTRK2** ]  gene expression have been detected at the time of primordial follicle formation.  Ref: 11836338 J Clin Endocrinol Metab, 2002
- Role of neurotrophins and neurotrophins receptors in the in vitro invasion and heparanase production of human prostate cancer cells.The role of the neurotrophins and their corresponding receptors TrkA, TrkB  [ **NTRK2** ] , TrkC, and p75NTR  [ **NGFR** ]  in neoplasia has received relatively little attention.  Ref: 10545017 Clin Exp Metastasis, 1999
- A sequential appearance of trkB  [ **NTRK2** ] .T1, the low affinity neurotrophin receptor p75  **NGFR** , and trkB  [ **NTRK2** ] .T2 in epithelial cochlear cells correlated with the formation of the inner sulcus of the organ.  Ref: 10494076 J Comp Neurol, 1999
- Differential expression of trkB  [ **NTRK2** ] .T1 and trkB  [ **NTRK2** ] .T2, truncated trkC, and p75  **NGFR**  in the cochlea prior to hearing function.  Ref: 10494076 J Comp Neurol, 1999
- Antigen blot analysis showed the presence of TrkA, TrkB  [ **NTRK2** ]  and p75NTR  [ **NGFR** ]  receptors in the visual cortex.  Ref: 9753104 Eur J Neurosci, 1998
- Activation of the TrkB  [ **NTRK2** ]  and the p75NTR  [ **NGFR** ]  by the addition of BDNF facilitates neuronal commitment and marked neurite genesis.  Ref: 12589813 Biochem Biophys Res Commun, 2003
- Mutants in the BDNF receptor gene trkB  [ **NTRK2** ]  and antibodies to its second receptor p75NTR  [ **NGFR** ]  have been used to determine the receptors and cells involved in this response.  Ref: 10995833 J Neurosci, 2000
- In contrast, the expression of TrkB  [ **NTRK2** ]  was down regulated and the expression of p75NTR  [ **NGFR** ]  was up regulated in prostate cancer after hormonal therapy.  Ref: 11442674 Int J Urol, 2001
- We have used immunofluorescence techniques with a panel of antibodies against known neurotrophin receptors trk A, trk B  [ **NTRK2** ] , trk C, p75NTR  [ **NGFR** ]  to map the locations of these receptors in the developing neuromuscular system of mice from our neurotrophin 3 NT 3 knockout colony.  Ref: 12141445 Int J Dev Biol, 2002
